# Exposure to oil pollution and maternal outcomes: The Niger Delta prospective cohort study

**DOI:** 10.1371/journal.pone.0263495

**Published:** 2022-03-02

**Authors:** Onome B. Oghenetega, Michael A. Okunlola, Godson R. E. E. Ana, Oludare Morhason-Bello, Oladosu A. Ojengbede

**Affiliations:** 1 Reproductive Health Science, Pan African University, Institute of Life and Earth Sciences (Including Health and Agriculture), University of Ibadan, Ibadan, Nigeria; 2 Department of Obstetrics and Gynecology, College of Medicine, University College Hospital, University of Ibadan, Ibadan, Nigeria; 3 Department of Environmental Health, Faculty of Public Health, University of Ibadan, Ibadan, Oyo State, Nigeria; London School of Hygiene and Tropical Medicine, UNITED KINGDOM

## Abstract

**Background:**

Maternal exposure to oil pollution is an important public health concern. However, there is a dearth of literature on the effects of maternal exposure to oil pollution on maternal outcomes in the Niger Delta region of Nigeria. This study was therefore designed to determine the effect of maternal exposure to oil pollution on maternal outcomes in the Niger Delta region of Nigeria.

**Methods:**

Prospective cohort study design involving 1720 pregnant women followed from pregnancy to delivery was conducted. The participants were 18–45 years old at a gestational age of less than 17 weeks, who attended randomly selected health facilities in the areas with high exposure and low exposure to oil pollution in the Niger Delta, Nigeria. Data were collected using an interviewer-administered questionnaire and review of medical records from April 2018 to April 2019. Multivariate log-binomial model was used to examine the effect of maternal exposure to oil pollution on the risk of adverse maternal outcomes adjusting for sociodemographic, maternal and lifestyle characteristics.

**Results:**

A total of 1418 women completed the follow-up and were included in the analysis. Women in high exposure areas had a higher incidence of premature rupture of membrane (PROM), caesarean section (CS) and postpartum haemorrhage (PPH) compared to women in areas with low exposure to oil pollution. After adjusting for cofounders, women in high exposure areas also had a higher risk of PROM (ARR = 1.96; 95% CI: 1.24–3.10) and PPH (ARR = 2.12; 95% CI: 1.28–3.36) in Model I–III when compared to women in areas with low exposure to oil pollution. However, pregnancy-induced hypertension and CS had no association with maternal exposure area status to oil pollution.

**Conclusion:**

Women in high exposure areas are at a higher risk of PROM and PPH. This calls for policies and intervention toward reducing maternal exposure to oil pollution in the Niger Delta region of Nigeria.

## Introduction

Maternal exposure to oil pollution is an important public health concern in the Niger Delta region of Nigeria. The region is rated as one of the most oil spill vulnerable areas in the world with about 123 gas flaring sites [1–4]. Several oil facilities are located close to the homes, farmlands, and water sources of host communities in this region [1]. Environmental pollutants such as volatile organic compounds (VOCs), heavy metals, polycyclic aromatic hydrocarbons (PAHs) are released when oil is spilt, and gas flared [1]. Several authors have reported that living in areas polluted by oil have adverse effects on human health [5–11].

A previous study has reported that oil pollution in the Niger Delta affects men and women disproportionately, with women being more exposed and vulnerable due to some cultural and socio–economic factors [12]. Food and Agriculture Organisation [13] showed that women are often poorer, more uneducated, possess fewer livelihood assets and depend more on natural resources for their livelihood. As such, they bear the heaviest burden of environmental degradation, especially pregnant women who are often considered a vulnerable population during disasters, both natural and chemical [14].

Oil pollution is known to be a predictor of several pathological conditions such as cancer, neurotoxicity, high blood pressure; and could lead to adverse maternal outcomes such as increased rate of miscarriage, intrauterine growth restriction, preterm birth, birth defect, low birth weight [15–17]. Furthermore, the integration of existing research revealed that oil pollution increases the risk of preterm birth, miscarriage, birth defects and gestational diabetes among women in oil polluted communities [16, 18, 19]. Several epidemiological studies have examined the association between exposure to oil pollutants and pregnancy outcomes. However, most of these studies are limited to developed regions of the world [18–21]. As such, little is known of the effects of maternal exposure to oil pollution on pregnancy outcomes in developing nations and in particular, in the oil polluted Niger Delta region of Nigeria.

Gay *et al*. [22] recommended that the effects of oil pollution on pregnancy outcomes should be studied in the Niger Delta region of Nigeria after a careful compilation of scientific research on the health effects of oil contamination in Deepwater Horizon, Ecuador, Exxon-Valdez, Kuwait, and Nigeria. Therefore, the Niger Delta area being one of the most oil polluted land in history presents a tremendous opportunity for prospective research that would address the pitfalls of previous works by investigating maternal residential exposure to oil pollution on adverse pregnancy outcomes. Currently, there is a dearth of literature on the effects of maternal residential exposure to oil pollution on adverse maternal outcomes in the Niger Delta of Nigeria.

## Methods

### Study area

The study was conducted in the Niger Delta region of Nigeria. The region consists of 9 crude oil-producing states with an estimated population of about 28 million people residing in about 3000 communities [23]. The Niger Delta region contains considerable oil reserves that have made the region the active hub for oil extraction and processing in Nigeria for the past 50 years [24]. The region is plagued with oil related contamination and has been rated as one of the most oil spill vulnerable areas in the world [1–3]. An estimated 10 to 13 million tons of hydrocarbons have been reportedly spilt into the Niger Delta over the last 50 years [25, 26]. During this period, over 77% of spilt hydrocarbons were not recovered [25, 26]. These spills and recurrent gas flaring have consequently led to contaminated air, water and land in the Niger Delta region, which has resulted in significant impacts on human health and ecological systems [27, 28].

### Study design, population and eligibility criteria

A prospective cohort design was employed in this study. All the pregnant women between the ages of 18 to 45 years who visited public health institutions for antenatal care in the selected public health facilities of Niger Delta, Nigeria constituted the study population. We restricted the study to pregnant women with a gestational age of less than 17 weeks, who have been residing in the selected areas for at least 3 years, and are willing to participate in the study including the follow-up period.

### Sample size and sampling procedure

The sample size of this study was determined using the double population proportion formula. The assumptions considered in the sample size calculation were a two‐sided confidence level of 95%, 80% power, and the ratio of high exposure areas to low exposure areas of 1 to 1. This study also considered a design effect of 2 because of the multistage sampling technique that was used, and a loss to follow up rate of 10%. The proportion of miscarriage among women living close to the oil field was obtained from a similar study [18]. The final sample size was 1720 pregnant women (860 women in high exposure areas and 860 women in low exposure areas).

Multistage sampling technique was used to select a representative of women in high exposure areas and women in areas with low exposure to oil pollution in the Niger Delta, Nigeria. Out of the nine (9) states (Akwa Ibom, Rivers, Delta, Bayelsa, Cross River, Edo, Imo, Ondo and Abia) in the region, Delta and Rivers States were purposively selected as states with high exposure to oil pollution due to the high production activities of crude oil, high crude oil spill record, and the presence of crude oil refinery. Ondo and Edo were also purposively selected as states with low exposure due to low production activities and low oil spill record and absence of oil refineries. These selections were informed by the record of the Nigerian Oil Spill Monitor, the monitor gives the public access to current official data on oil spills collected by the National Oil Spill Detection and Response Agency (NOSDRA) and the National Bureau of Statistic. The four states were stratified into oil producing and non-oil producing areas. Two health facilities serving oil producing areas in Delta and Rivers State were randomly selected. For the reference group, two hospitals/health facilities serving non-oil producing areas in Ondo State and Edo State were also randomly selected for the study. Finally, random sampling technique was used to select the study participants.

#### Study instrument, quality assurance, and pre-test

A structured and interviewer-administered questionnaire was prepared by reviewing several related literature and related international guidelines [5, 9, 19, 29, 30]. It was organised into several sections, such as socio-demographic characteristics (maternal age, marital status, religion, level of education, mothers’ occupations, household income and main source of cooking fuel), maternal and lifestyle characteristics (mid upper arm circumference, gravidity, previous miscarriage, previous stillbirth, previous infant death, alcohol intake, smoking and diet diversity status); oil pollution exposure characteristics (exploration activities, oil spill incidence, gas flaring incidence, perception on air quality, perception on water quality, perception on soil quality); and adverse maternal (PIH, PROM, PPH, and caesarean delivery). The data collection and supervision of the study were handled by experienced research assistants and professionals (midwives or nurses). Data collectors and supervisors received five (5) days of training before the data collection period began. The training covered the study’s objectives, ethical considerations, interviewing techniques, study inclusion criteria, follow-up procedures, and the overall content of the data collection instrument. The data collection instrument was validated using face and content validity. Pre-test of the data collection instrument was conducted before the actual data collection. We used feedback from the pilot to modify the questions.

### Data collection procedure

A pre-tested structured and interviewer-administered questionnaire was used for data collection. It was organised into several sections, such as socio-demographic characteristics, maternal and lifestyle characteristics and adverse maternal outcomes.

Data regarding sociodemographic characteristics, maternal and lifestyle characteristics were collected at the first encounter with study participants in the antenatal clinic. Data on adverse maternal outcomes were collected from pregnant women that were followed prospectively from their index pregnancy to 24 hours after delivery. The follow-up period was from April 2018 to April 2019. Missing data were gathered from the woman’s antenatal and other medical records. The completeness and accuracy of the collected data were checked daily during the data collection period by the research supervisors and the principal investigator.

### Definition of outcomes

Adverse neonatal outcomes were measured as the occurrence of Pregnancy—induced hypertension (PIH), Premature rupture of membrane (PROM), Caesarean section or delivery (CS) and Postpartum haemorrhage (PPH). PIH is defined as systolic blood pressure ≥ 140mmHg and/ or diastolic blood pressure ≥ 90 mmHg in a previously normotensive pregnant woman who is ≥ 20 weeks of gestation and with or without proteinuria [31]; PROM is defined as the rupture of the foetal membrane after 37 weeks of gestation prior to the onset of labour [32]; CS is defined as a surgical procedure in which a foetus is delivered through abdominal and uterine incision [33], and PPH is defined as blood loss of 500ml or more within 24hours after birth [34].

### Definition of exposure variable

The primary exposure was women in high exposure areas to oil pollution. Women in high exposure areas is defined as women who have resided for at least three (3) years in oil producing areas of states in the Niger Delta with high crude oil production activities, high incidence of oil spills and presence of a crude oil refinery. Women in low exposure areas is defined as women who have resided for at least three (3) years in non-oil producing communities of states in the Niger Delta with low crude oil production activities, low incidence of oil spills and absence of a crude oil refinery.

### Definition covariates

Socio-demographic characteristics included maternal age that was categorised as <25 years, 25–34 years and ≥ 35 years; educational status as tertiary or non-tertiary; monthly household income in naira as < 50,000 or ≥ 50,000; marital status as (married, single and others); religion as (Christian, Muslim, or others); Mother’s occupation as (non-oil and gas related, or oil and gas related); and main source of cooking fuel as (clean or unclean). Women who used gas or electricity as their main source of cooking fuel were classified as clean fuel users, while women that used firewood, kerosene, charcoal, or crop residue/sawdust as their main source of cooking fuel were classified as unclean fuel users [35].

Maternal and lifestyle characteristics included gravidity categorised as primigravida or multigravida; previous miscarriage as (yes or no); previous stillbirth as (yes or no); and previous infant death—the death of a baby before the first birthday—as (yes or no); smoking as (yes or no); alcohol intake as (yes or no); mid upper arm circumference (MUAC) as MUAC < 28cm or MUAC ≥ 28cm. Women with MUAC < 28cm were considered to be normal and women with MUAC ≥ 28cm were considered to be pre-gestational overweight and/or obese [36]. Women’s diet diversity status was measured using the standardised tools for women diet diversity score based on the FAO guidelines for measuring minimum dietary diversity score in women with the consumption of ten food items within a period of 24 hours [37]. Women who consumed less than five food items and greater than or equal to 5 food items were classified to have inadequate diet diversity status and adequate diet diversity status respectively [37].

### Statistical analysis

Data were entered and cleaned using SPSS (version 23.0; IBM) software and analysed using STATA (version 14.0; StataCorporation) software. Descriptive statistics like frequencies and summary statistics (mean, standard deviation (SD), and percentage) were used to describe the participants’ characteristics. Categorical data were compared using Pearson’s chi-square test, and independent t-test was used for comparison of the mean difference.

Log-binomial model was used to determine the relative risk summary metric for the associations between exposure to oil pollution and adverse pregnancy outcomes and to control the effect of potential confounders. Log-binomial model is a special case of a generalised linear model, specifically applying a log link function to binomial outcome data for modelling adjusted relative risk in prospective data [38, 39]. The public health research community have suggested the use of relative risk (RR) for cohort study instead of odds ratio. This can be attributed to the difficulty in interpreting odds ratio, as it is sometimes misinterpreted as relative risk [39].

Separate log-binomial models were tested and presented for each outcome. Variables were included in the multivariable log-binomial model based on literature review and their association with each adverse maternal outcome (p-value ≤ 0.20) in the bivariate analysis. Crude relative risk (CRR) was generated in model I. In model II, the adjusted relative risk (ARR) for the associations between exposure to oil pollution and adverse pregnancy outcomes were determined after controlling for sociodemographic characteristics (maternal age, level of education, mother’s occupation, household income, and source of cooking fuel). In model III, the association between exposure to oil pollution and pregnancy outcomes were adjusted for sociodemographic characteristics plus maternal and lifestyle variables (MUAC, gravidity, smoking, and diet diversity status). Variables in each model were mutually adjusted for each other. Moreover, multicollinearity between the variables was checked using the variance inflation factor (VIF). Finally, statistical significance was established at ARR≠ 1 with a 95% CI and P-value ≤ 0.05.

### Ethical consideration

The study was approved by the ethics committee of the Institute for Advanced Medical Research and Training (IAMRAT), College of Medicine, University of Ibadan, Ibadan, Nigeria with the UI/UCH EC Registration Number of NHREC/05/01/2008a and UI/UCH Ethics Committee assigned number, UI/EC/17/0517. In addition, the study was also approved by the Ethics Committee of Rivers, Ondo, Edo and Delta States Hospital Management Board. Permission for the use of the facility was obtained from the head of each selected health facility. Written informed consent was obtained from each participant; after explaining the purpose of the study, benefits and risks. The right to participate or withdraw from participation was also made explicit to them to ensure that participation was voluntary and to make them feel free from coercion or pressure.

## Results

**Fig 1:**
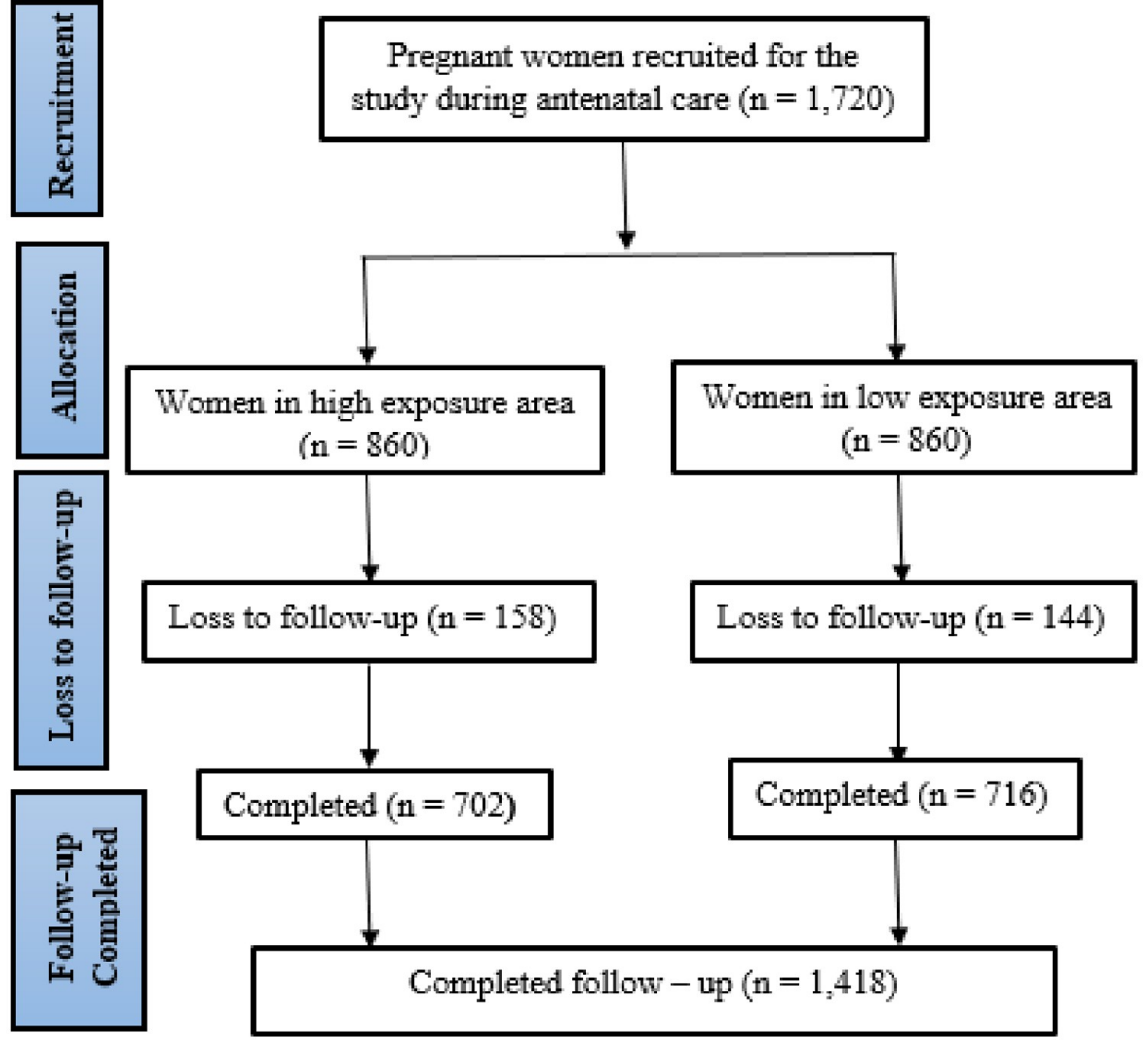
A flow diagram of study participants.

A total of 1720 women were recruited at the ANC clinic and followed from pregnancy to delivery. Overall, a total of 1418 (82.4%) of the women completed the study. Based on exposure, a total of 702 (81.6%) women in high exposure areas and 716 (83.3%) women in low exposure areas completed the study as presented in Fig 1.

The result of sociodemographic characteristics as presented in Table 1 shows that women in high (29.52 ± 5.59 years) and low (29.82 ± 4.92 years) exposure areas had almost the same mean age (p = 0.227). Majority of the women in high exposure areas 76.2% (n = 533) and low exposure areas 92.7% (n = 664) were married. More than half 57.3% (n = 410) of the women in low exposure areas had tertiary education while only 33.2% (n = 254) had acquired tertiary education among women in high exposure areas. Over 95% of the women in high and low exposure areas to oil pollution professed Christianity.

**Table 1:** Sociodemographic characteristics of women by exposure areas in the Niger Delta.

Variable	Total participants (n– 1,418)	High exposure areas n (%)	Low exposure areas n (%)	P–value
**Maternal age**				
< 25years	237 (16.7%)	137 (19.5%)	100 (14.0%)	0.006
25–34 years	893 (63.0%)	416 (59.3%)	477 (66.6%)	
≥ 35 years	288 (20.3%)	149 (21.2%)	139 (19.4%)	
Mean ± SD	29.69 ± 5.26 years	29.52 ± 5.59 years	29.82 ± 4.92 years	0.227
**Marital status**				
Married	1197 (84.4%)	533 (75.9%)	664 (92.7%)	< 0.001
Single and others	221 (15.6%)	169 (24.1%)	52 (7.3%)	
**Religion**				
Christian	1368 (96.5%)	681 (97.0%)	687 (95.9%)	0.136
Muslim	43 (3.0%)	16 (2.3%)	27 (3.8%)	
Others	7 (0.5%)	5 (0.7%)	2 (0.3%)	
**Level of education**				
Tertiary	643 (45.3%)	233 (33.2%)	410 (57.3%)	< 0.001
Non-tertiary	775 (54.7)	469 (66.8%)	306 (42.7)	
**Mother’s occupation**				
Not oil and gas related	1370 (96.6%)	665 (94.7%)	705 (98.5%)	< 0.001
Oil and gas related	48 (3.4%)	37 (5.3%)	11 (1.5%)	
**Household income (N)**				
< 50,000	356 (25.1%)	202 (28.8%)	154 (21.5%)	0.002
≥ 50,000	1062 (74.9%)	500 (71.2%)	562 (78.5%)	
**Main source of cooking fuel**				
Clean	867 (61.2%)	349 (48.7%)	518 (73.9%)	< 0.001
Unclean	550 (38.8%)	367 (51.3%)	183 (26.1%)	

Furthermore, 5.3% (n = 37) of women in high exposure areas were into oil and gas related occupations compared to 1.5% (n = 11) of women in low exposure areas that were into oil and gas related occupations. Higher proportion of women, 28.8% (n = 202) in high exposure areas had household monthly income below ₦50,000 compared to 21.5% (n = 154) in low exposure (p = 0.002) areas. The major sources of cooking fuel among the women showed that 48.7% (n = 349) of the women in high exposure areas used clean fuel sources compared to 74% (n = 579) of the women in low exposure areas who used clean fuel sources (p = <0.001).

Table 2 shows that about half of the pregnant women in the high exposure areas agreed to oil exploration activities, spillage and gas flaring incidents within and around their communities compared to less than 10% of the pregnant women in low exposure areas. Also, a higher proportion of pregnant women in the high exposure areas consented to the contamination of air (p = <0.001), water (p = <0.001), and soil (p = <0.001) by oil pollution within and around their communities when compared with pregnant women in the low exposure areas.

**Table 2:** Oil pollution exposure characteristics by exposure areas in the Niger Delta.

Variable	Total participants (n– 1,418)	High exposure areas n (%)	Low exposure areas n (%)	P–value
**Exploration activities**				
No	948 (66.9%)	269 (38.3%)	679 (94.8%)	<0.001
Yes	470 (33.1%)	433 (61.7%)	37 (5.2%)	
**Oil spill incidents**				
No	1044 (73.7)	362 (51.6%)	683 (95.4%)	<0.001
Yes	373 (26.3%)	340 (48.4%)	33 (4.6%)	
**Gas flaring incidents**				
No	1022 (72.1%)	375 (53.4%)	647 (90.4%)	<0.001
Yes	395 (27.9%)	327 (46.6%)	69 (9.6%)	
**Perception on air quality**				
Not contaminated	693 (48.9%)	193 (27.3)	500 (69.8)	<0.001
Slightly contaminated	493 (34.8)	306 (43.7)	187 (26.2%)	
Contaminated	232 (16.3%)	203 (29.0%)	29 (4.1%)	
**Perception on water quality**				
Safe not contaminated	1023 (72.1%)	383 (54.6%)	640 (89.4%)	<0.001
Safe but slightly contaminated	299 (21.1%)	239 (34.0%)	60 (8.4%)	
Unsafe and contaminated	96 (6.8%)	80 (11.4%)	16 (2.2%)	
**Perception on soil quality**				
Not contaminated	888 (62.6%)	298 (42.5%)	590 (82.4%)	<0.001
Slightly contaminated	421 (29.7)	305 (43.4%)	116 (16.2%)	
Contaminated	109 (7.7%)	99 (14.1%)	10 (1.4%)	

On maternal variables as presented in Table 3, 69.4% (n = 456) and 35.6% (n = 250) of the women in high exposure areas had MUAC ≥ 28cm and are primigravida compared to 69.0% (n = 479) and 39.8% (n = 285) of women in low exposure areas who had MUAC ≥ 28cm and are primigravida, respectively. Furthermore, 17.8% (n = 125) of women in high exposure areas had previously experienced miscarriage compared to 18.0% (n = 129) of women in low exposure areas (p = 0.918). Also, a higher proportion of women in high exposure areas 8.4% (n = 59) had experienced stillbirth compared to 4.6% (n = 33) of women in low exposure areas (p = 0.004).

**Table 3:** Maternal and lifestyle characteristics of women who completed follow-up study by exposure areas in the Niger Delta.

Variable	Total participants (n– 1,418)	High exposure areas n (%)	Low exposure areas n (%)	P–value
**MUAC**				
MUAC < 28cn	416 (30.8%)	201 (30.6%)	215 (31.0%)	0.878
MUAC ≥ 28cm	935 (69.2%)	456 (69.4%)	479 (69.0%)	
**Gravidity**				
Primigravida	535 (37.7%)	250 (35.6%)	285 (39.8%)	0.103
Multigravida	883 (62.3%)	452 (64.4%)	431 (60.2%)	
**Previous miscarriage**				
No	1164 (82.1%)	577 (82.2%)	587 (82.0%)	0.918
Yes	254 (17.9%)	125 (17.8%)	129 (18.0%)	
**Previous stillbirth**				
No	1326 (93.5%)	643 (91.6%)	683 (95.4%)	0.004
Yes	92 (6.5%)	59 (8.4%)	33 (4.6%)	
**Previous infant death**				
No	1327 (93.6%)	641 (91.3)	686 (95.8)	P<0.001
Yes	91 (6.4%)	61 (8.7)	30 (4.2)	
**Alcohol intake**				
No	1094 (77.2%)	531 (75.6%)	563 (78.6%)	0.180
Yes	324 (22.8%)	171 (24.4%)	153 (21.4%)	
**Smoking**				
No	1357 (95.7%)	662 (94.3%)	695 (97.1%)	0.010
Yes	61 (4.3%)	40 (5.7%)	21 (2.9%)	
**Diet diversity status**				
Adequate	797 (56.2%)	370 (52.7%)	427 (59.6%)	0.009
Inadequate	621 (43.8%)	332 (47.3%)	289 (40.4%)	

Also, lifestyle variables as presented in Table 3 shows that 24.4% (n = 171) and 5.7% (n = 40) of women in high exposure areas agreed to alcohol consumption and cigarette smoking compared to 21.4% (n = 153) and 2.9% (n = 21) of women in low exposure areas who agreed to alcohol consumption and cigarette smoking, respectively. Moreover, almost half of the women 47.3% (n = 332) in high exposure areas to oil pollution had inadequate diet diversity compared to 40.4% (n = 289) of women in low exposure areas (p = 0.009).

**Fig 2:**
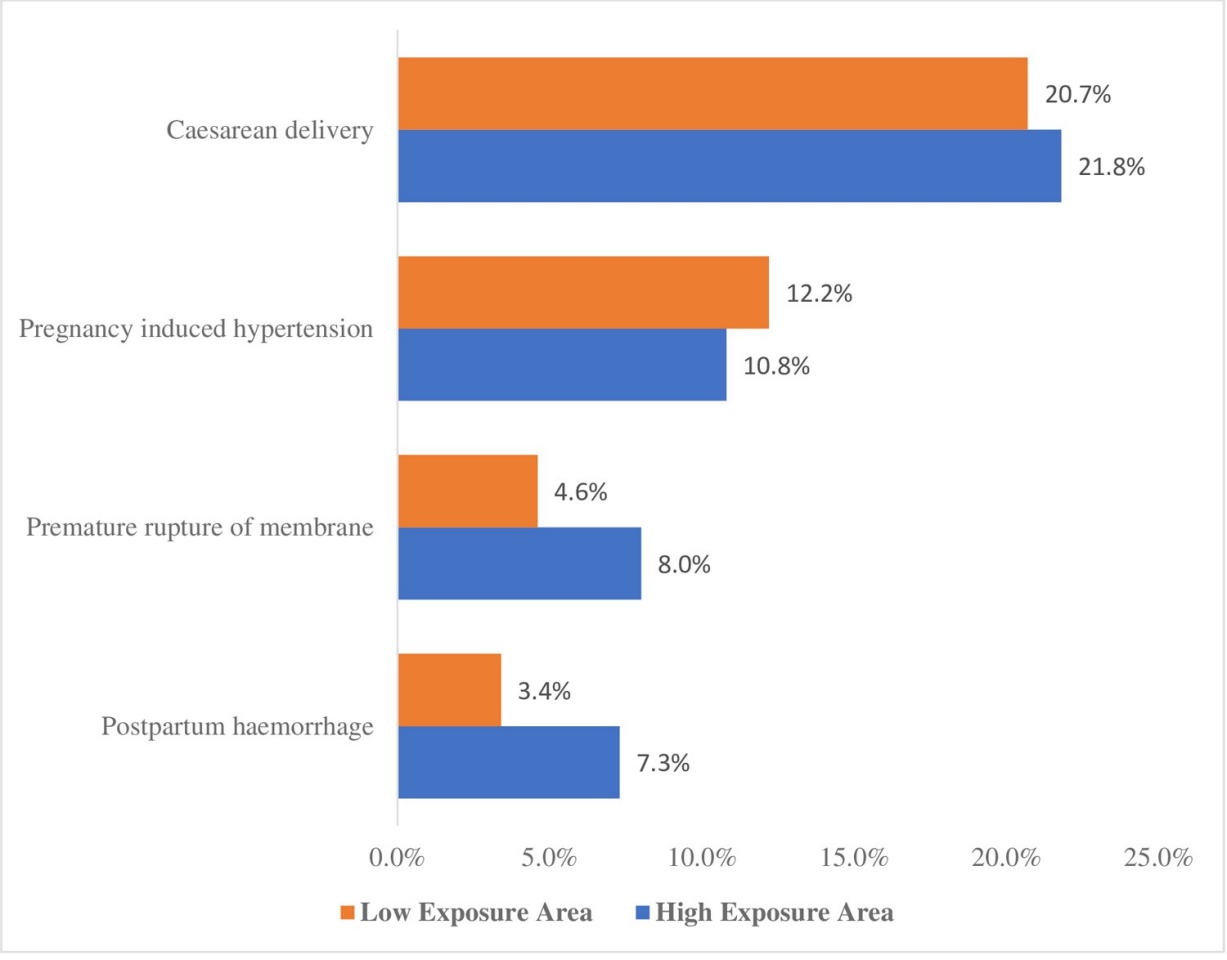
Incidence of adverse maternal outcomes due to maternal exposure areas to oil pollution.

Incidence of adverse maternal outcomes due to exposure to oil pollution as presented in Fig 2 shows that the incidence of PROM was higher among women in high exposure areas than women in low exposure areas (8.0% vs 4.6%). Similarly, PPH was higher among women in high exposure areas than among women in low exposure areas to oil pollution (7.3% vs 3.4%). In addition, 21.8% of women in high exposure areas to oil pollution and 20.7% of women in low exposure areas had Caesarean delivery. However, pregnancy-induced hypertension was slightly lower among women in high exposure areas compared to women in low exposure areas (10.8% vs 12.2%).

The association between maternal exposure to oil pollution and women who had PROM is presented in Table 4. The unadjusted log-binomial model (model 1) shows that only high exposure to oil pollution (CRR = 1.72; 95% CI: 1.14–2.62) has a significant association with women who had PROM at the crude level. In multivariate model II, after adjusting for socio-demographic variables, the results showed that only high exposure to oil pollution had a significant association with PROM (ARR = 1.99; 95% CI: 1.27–3.12). After adjusting for sociodemographic characteristics plus maternal and lifestyle characteristics (Model III), high exposure to oil pollution (ARR = 1.96; 95% CI: 1.24–3.10) was still significantly associated with PROM.

**Table 4:** Log-binomial regression (Models I-IV) showing the association of oil pollution exposure and PROM.

Variables	PROM		Model I	Model II	Model III
	Yes, n (%)	No, n (%)	CRR (95% CI)	ARR (95% CI)	ARR (95% CI)
**Exposure areas**					
Low	33 (4.6)	682 (95.4)	1	1	1
High	56 (8.0)	646 (92.0)	1.72 (1.14–2.62)*	1.99 (1.27–3.12)*	1.96 (1.24–3.10)*
**Maternal age (years)**					
>25	14 (5.9)	222 (94.1)	1	1	1
25–34	54 (6.0)	840 (94.0)	1.02 (0.58–1.80)	1.02 (0.57–1.82)	1.00 (0.54–1.86)
≥ 35	21 (7.3)	266 (92.7)		1.17 (0.60–2.29)	1.07 (0.50–2.27)
**Level of education**					
Tertiary	42 (6.6)	599 (93.4)	1	1	1
Non-tertiary	47 (6.1)	729 (93.9)	0.92 (0.62–1.38)	0.77 (0.50–1.19)	0.74(0.46–1.15)
**Mother’s occupation**			1.34 (0.51–3.51)	1.20 (0.45–3.17)	1.30 (0.49–3.45)
Non-oil and gas related	85 (6.2)	1284(93.8)			
Oil and gas related	4 (8.3)	44 (91.7)			
**Household income (N)**					
< 50,000	25 (7.0)	333 (93.0)	1	1	1
≥ 50,000	64 (6.0)	995 (94.0)	0.87 (0.55–1.35)	0.85 (0.54–1.35)	0.79 (0.50–1.26)
**Sources of cooking fuel**					
Clean sources	50 (5.8)	816 (94.2)	1	1	1
Unclean sources	39 (7.1)	512 (92.9)	1.23 (0.82–1.84)	1.47 (0.96–2.23)	1.48 (0.97–2.28)
**MUAC**				-	
MUAC < 28cm	26 (6.3)	389 (93.7)	1		1
MUAC ≥ 28cm	59 (6.3)	875 (93.7)	1.01 (0.65–1.58)		1.01 (0.65–1.59)
**Gravidity**				-	
Primigravida	35 (6.5)	502 (93.5)	1		1
Multigravida	54 (6.1)	826 (93.9)	0.94 (0.62–1.42)		0.84(0.53–1.33)
**Smoking**				-	
No	85 (6.3)	1271 (93.7)	1		1
Yes	4 (6.6)	57 (93.4)	1.05 (0.40–2.76)		1.02 (0.39–2.68)
**Diet diversity status**				-	
Adequate	54 (6.8)	741 (93.2)	1		1
Inadequate	35 (5.6)	587 (94.4)	0.83 (0.55–1.25)		0.83 (0.54–1.26)

*p<0.05, CRR Crude relative risk, ARR Adjusted relative risk, CI Confidence interval.

Notes: Model I: shows crude relative risk. Model II: Adjusted for socio-demographic variables. Model III: Adjusted for socio demographic variables and maternal/lifestyle variables.

The bivariate log-binomial model of the association between oil pollution and PIH shows that high exposure to oil pollution has no significant association with PIH (CRR = 0.89; 95% CI: 0.67–1.19) and Caesarean delivery (CRR = 1.05; 95% CI: 0.86–1.29).

Moreover, the association between maternal exposure to oil pollution and postpartum haemorrhage is presented in Table 5. The unadjusted log-binomial model (Model 1) shows that high exposure to oil pollution (CRR = 2.17; 95% CI: 1.35–3.48) and maternal age ≥ 35 years (CRR = 2.02; 95% CI: 1.02–3.98) had a significant association with PPH at the crude level. In multivariate Model II, after adjusting for socio-demographic variables (maternal age, educational status, mother’s occupation, household income and source of cooking fuel), the results show that high exposure to oil pollution (ARR = 2.15; 95% CI: 1.30–3.57) and maternal age ≥ 35 years (ARR = 2.01; 95% CI: 1.01–4.00) remained significantly associated with PPH. In Model III, high exposure to oil pollution (ARR = 2.12; 95% CI: 1.28–3.58) and maternal age ≥ 35 years (ARR = 3.07; 95% CI: 1.34–7.04) remained significantly associated with PPH after adjusting for sociodemographic variables plus maternal and lifestyle variables.

**Table 5:** Log-binomial regression (Models I–IV) showing the association of oil pollution exposure and PPH.

Variables	PPH		Model I	Model II	Model III
	Yes, n (%)	No, n (%)	CRR (95% CI)	ARR (95% CI)	ARR (95% CI)
**Exposure status**					
Low	24 (3.4)	692 (96.6)	1	1	1
High	51 (7.3)	651 (92.7)	2.17 (1.35–3.48)*	2.15 (1.30–3.57)*	2.12 (1.28–3.56)*
**Age**					
>25	11 (4.7)	225 (95.3)	1	1	1
25–34	37 (4.1)	858 (95.9)	0.89 (0.46–1.71)	0.89 (0.45–1.74)	1.21 (0.57–2.58)
≥ 35g	27 (9.4)	260 (90.6)	2.02 (1.02–3.98)*	2.01 (1.01–4.00)*	3.07 (1.34–7.04)*
**Education**					
Tertiary	34 (5.3)	607 (94.7)	1	1	1
Non-tertiary	41 (5.3)	736 (94.7)	1.00 (0.64–1.55)	0.82 (0.52–1.31)	0.84 (0.52–1.35)
**Occupation**					
Non-oil and gas related	70 (5.1)	1300 (94.9)	1	1	1
Oil and gas related	5 (10.4)	43 (89.6)	2.03 (0.86–4.81)	2.07 (0.86–4.98)	2.14 (0.89–5.16)
**Household income (N)**					
< 50,000	22 (6.1)	336 (93.9)	1	1	1
≥ 50,000	53 (5.0)	1007(95.0)	0.81 (0.50–1.32)	0.82 (0.50–1.34)	0.80 (0.48–1.32)
**Cooking fuel**					
Clean	49 (5.7)	817 (94.3)	1	1	1
Unclean	26 (4.7)	526 (95.3)	0.83 (0.52–1.32)	1.05 (0.65–1.69)	1.01 (0.62–1.65)
**MUAC**				-	
MUAC < 28cm	23 (5.5)	392 (94.5)	1		1
MUAC ≥ 28cm	48 (5.1)	887 (94.9)	0.93 (0.57–1.50)		0.89 (0.55–1.44)
**Gravidity**				-	
Primigravida	30 (5.6)	507 (94.4)	1		1
Multigravida	45 (5.1)	836 (94.9)	0.91 (0.58–1.43)		0.66 (0.39–1.10)
**Smoking**				-	
No	72 (5.3)	1258 (94.7)	1		1
Yes	3 (4.9)	58 (95.1)	0.93 (0.30–2.86)		0.90 (0.29–2.78)
**Diet diversity**				-	
Adequate	46 (5.8)	750 (94.2)	1		1
Inadequate	29 (4.7)	593 (95.3)	0.81 (0.51–1.27)		0.78 (0.50–1.25)

*p<0.05, CRR Crude relative risk, ARR Adjusted relative risk, CI Confidence interval.

Notes: Model I: shows crude relative risk. Model II: Adjusted for socio-demographic variables. Model III: Adjusted for socio demographic variables and maternal/lifestyle variables.

## Discussion

In this prospective study of pregnant women in the Niger Delta of Nigeria, women residing in areas with high exposure to oil pollution have a higher incidence and risk of PPH and PROM compared to women residing in areas with low exposure. However, level of exposure to oil pollution was not associated with the incidence of pregnancy induced hypertension and caesarean delivery in the study area. The higher incidence of PROM among women in high exposure areas was 1.4 times lower than a study conducted in China among women exposed to airborne particulate matter [40] and 1.5 times lower in another study also conducted in China among women exposed to lead [41]. However, it was higher than the prevalence rate reported in the USA among women exposed to air pollution [42]. Women in high exposure areas were about 2 times at high risk of PROM compared to women in low exposure areas in this study even after adjusting for potential confounders. This is consistent with previous studies that have shown that women exposed to environmental pollutants are at high risk of PROM [41, 42]. The increased risk of PROM could be possibly due to inflammatory and oxidative stress pathways. Elevation of proinflammatory cytosines due to exposure to environmental pollutants alters the membrane barrier function and thus, leads to PROM [41, 43]. On oxidative stress pathway, exposure to oil pollutants (such as PAH, Lead, CO, SO_2_) could induce oxidative stress damaging DNA and causing the release of destructive enzymes, consequently damaging the collagen of the foetal membrane leading to PROM [41, 42, 44, 45]. PROM has been associated with an increased incidence of pregnancy related morbidity and mortality [32, 46]. Thus, this study supports the campaign for early antenatal care for women in the Niger Delta region and optimum obstetric care for women with PROM in the oil-polluted communities which are pivotal in reducing its adverse consequences.

Although the incidence of Caesarean delivery was higher among women residing in areas with high exposure to oil pollution than women residing in low exposure areas, the bivariate analysis (Model 1) shows a non-significant association (CRR = 1.05; 95% CI: 0.86–1.29). This is consistent with a study conducted in Ghana among women exposed to polluting cooking fuel [35]. Scholars have shown that most women prefer spontaneous vaginal delivery, however, there has been a steady increase in Caesarean section globally [47, 48]. The steady rise has been attributed to certain sociodemographic characteristics such as older age, rurality, previous caesarean delivery and the fear of labour pains as well as life threatening obstetric conditions such as cephalic-pelvic disproportion among others [47, 49, 50]. Also, a non-significant association was observed between PIH and exposure status to oil pollution in this study. This is in line with the study conducted in Southern Louisiana among women who were pregnant both before and after the oil spill that reported no significant association between hypertension disorder of pregnancy and oil pollution [19], and a study in Ghana among women exposed to polluting cooking fuel [35]. Despite extensive reports of anxiety disorder, depression and/or environmental apprehension among residents of oil polluted communities [51, 52], women in high exposure areas had a lower incidence of PIH when compared to those in low exposure areas which negate a priori expectation, and contradicted the findings of Ezejimofor et al. [15] who posited a five times likelihood of hypertensive disorder among persons in high exposure areas. The difference between the findings of this study and Ezejimofor et al. [15] could be attributed to the difference in study design, sample size, sociodemographic, maternal and lifestyles factors.

The incidence of PPH was twice higher among women in high exposure areas compared to women in low exposure areas in this study. This implies that women in areas with high exposure to oil pollution are more prone to PPH. The incidence among women in areas with high exposure in this study was about two times higher than that reported among women using polluting cooking fuel in Ghana [35] and almost five times higher among women in low resource settings in Zimbabwe [53]. Also, this was higher than the prevalence of PPH recorded in Plateau State [54] and Port Harcourt in Rivers State [55], but lower than the prevalence recorded in Delta State [56]. Moreover, the multivariate log-binomial model showed that women in high exposure areas had 2.12 times increased risk of developing PPH compared to women in low exposure areas. A plausible biological mechanism for this might be through the oxidative pathway that has been associated with inhibition uterine contraction and retained placenta [57, 58]. Increased air pollution from gas flaring in oil refineries (including illegal refineries) and oil spillage sites resulting in the release of pollutants such as polycyclic aromatic hydrocarbon and volatile organic hydrocarbon into the atmosphere have been associated with oxidative stress [59–61]. Also, studies have shown that oxidative stress can result in the inhibition of uterine contraction [57] and retained placenta [58]. Uterine atony and retained placenta are the leading causes of PPH [34, 53, 54]. The higher incidence of PPH among women in high exposure areas can be prevented or reduced by increased access to uterotonics in the Niger Delta. This study, therefore, strongly encourages the effective implementation of the Nigeria Federal Ministry of Health’s policy on the community-based distribution of misoprostol for PPH prevention especially in oil-polluted communities with a higher relative risk for PPH, which is pivotal to achieving maternal death reduction in the Niger Delta region of Nigeria.

The study was not without limitations. First, accurate assessment of individual level of exposure to oil pollutants was a major concern in this study. It was assumed that women residing within the same exposure areas have the same exposure status. Second, migration and unexpected travel of participants during the follow-up period from one exposure area to another without the knowledge of the principal investigator was also a limitation in the study. Third, the loss of respondents at the follow-up period might have introduced follow up bias. However, this limitation was accounted for during sample size calculation. Finally, this study could not control for all potential bias but the awareness of possible bias allowed thorough scrutiny of the results.

## Conclusions

This study reveals that women in areas with high exposure to oil pollution have a higher risk of PPH and PROM compared to women in low exposure areas. However, the incidence of PIH and CS have no association with maternal exposure areas in this study. This calls for policies and intervention toward reducing maternal exposure to oil pollution in the Niger Delta region of Nigeria.

## Supporting information

S1 File Data collection questionnaire. (DOCX)

S2 File Raw data set. (SAV)
