# Supplementary material for: Exposure to oil pollution and maternal outcomes: The Niger Delta prospective cohort study
Source: PLoS One. 2022 Mar 2;17(3):e0263495. doi: 10.1371/journal.pone.0263495 (PMC9090450; doi:10.1371/journal.pone.0263495)
Supplement: S1 File — (DOCX) [file pone.0263495.s001.docx]

**INTERVIEW SCHEDULE**

**PAN AFRICAN UNIVERSITY, LIFE AND EARTH SCIENCES INSTITUTE (PAULESI)**

**UNIVERSITY OF IBADAN, IBADAN**

| Questionnaire ID: |  |
| --- | --- |
| State: |  |
| Community |  |
| Health facility name: |  |
| Card /Phone number: |  |
| Date of interview: |  |
| Time of interview: |  |
| Have you stayed in the community for 3 years and above: Yes=1 No=2 |  |

**Section A: Socio-demographic Characteristics of respondents**

| **S.N** | **Questions** | **Response** |
| --- | --- | --- |
| 1. | How old are you (in years)? |  |
| 2. | Marital status: Single=1 Married=2 Cohabiting=3 Separated=4 widowed=5 |  |
| 3. | Household size (in persons) |  |
| 4. | Education of respondent: *Primary education=1 Secondary education=2 tertiary education=3* No formal education=4 5=vocational training *Others, please specify_______________* |  |
| 5. | Religion of respondent: *Christianity=1 Islam=2 Traditional=3 others, (specify) __________________* |  |
| 6. | Migration status of Respondent: *1= Native 2=immigrant 3*=*others, (specify) ___________* |  |
| 7. | Length of residence in the community (years) |  |
| 8. | Main occupation of respondent: *Farming=1 Fishing=2 Business=3 Civil servant=4 Oil and gas related occupation=5 Others, specify ____________* |  |
| 9. | If married, your husband’s occupation: *Farming=1 Fishing=2 Business=3 Civil servant=4 Oil and gas related occupation=5 Others, specify ____________* |  |
| 10 | Estimate your monthly income (Naira) | _______ |
| 11 | If married estimate your monthly income of husband (Naira) | ________ |
| 12 | Tick which of the following is available in your household  *Electricity ( ) A radio ( ) Television ( ) Mobile telephone ( ) Refrigerator ( ) Cable TV ( ) Generator ( ) Air conditioner ( ) Computer ( ) Electric iron ( ) fan ( )* |  |
| 13 | Main source of fuel used for cooking:  *Electricity=1 Gas=2 Charcoal=3 Firewood=4 Kerosene=5 Others, specify ____________* |  |

| \| **Section B: Oil Pollution exposure characteristics** \| \| \| \| --- \| --- \| --- \| \| **S/N** \| **Questions** \| **Response** \| \| 14 \| Is there oil exploration and extraction activities around your community of residence? *Yes=1 No=2* \|  \| \| 15 \| Is there gas flaring incidence around your community of residence? *Yes=1 No=2* \|  \| \| 16 \| Has there been oil spill incidence around your community of residence? *Yes=1 No=2* \|  \| \| 17 \| What do you think about the quality of the air in your community of residence: *Not contaminated=1* *Slightly contaminated=2 Contaminated=3* \|  \| \| 18 \| Perception of drinking water*: safe not contaminated=1* safe but s*lightly contaminated=2 unsafe and Contaminated=3 very unsafe and highly contaminated=4* \|  \| \| 19 \| What do you think about the quality of the soil in this community? *Not contaminated=1* *Slightly contaminated=2 Contaminated=3* \|  \|   **Section C. Past Obstetric History of Respondents** | | |
| --- | --- | --- | --- | --- | --- | --- | --- | --- | --- | --- | --- | --- | --- | --- | --- | --- | --- | --- | --- | --- | --- | --- | --- | --- | --- | --- |
|  | **Questions** | **Response** |
| 20 | What was the age at which you married for the first time? | _____ years |
| 21 | Have you ever been pregnant, other than this pregnancy? *Yes=1 No=2* |  |
| 22 | What was your age at your first pregnancy? | _____years |
| 23 | Have you ever had a miscarriage? *Yes=1 No=2* |  |
| 24 | If yes, how many times? |  |
| 25 | Have you given birth to a child? *Yes=1 No=2* |  |
| 26 | If yes to question 38, how many live births did you have? |  |
| 27 | Did you have stillbirths? *Yes=1 No=2* |  |
| 28 | If yes to question 40, how many stillbirth? |  |
| 29 | Have you experienced an infant death? |  |
| 30 | How many home deliveries had you had? | _______ |
| 31 | How many deliveries did you have in health facilities? | ______ |
| 32 | Have you given birth to any deformed child at all, then which part of the body was deformed? Limbs=1 Chest/Back=2 Sense Organ (ear, eyes, mouth)=3 None=4 |  |
| 33 | Do you have any incidence of such deformity in your parental family, then how many cases? One=1 Two=2 Three=3 More than three=4 None=5 |  |
| 34 | During your current pregnancy, did your husband/partner insult, humiliate, do something to scare you, or verbally hurt you? *Yes =1 No =2* |  |
| 35 | How often do you drink alcoholic beverages? *Never =1 Daily=2 Once or twice a week=3 Once a week or more=4 No response=5 Others (specify) ________* |  |
| 36 | Did you drink alcoholic beverages during this pregnancy? *Yes=1 No=2* |  |
| 37 | How often does someone smoke cigarettes around you? *Never =1 Daily=2 Once or twice a week=3 Once a week or more=4 No response=5 Others (specify) _____________* |  |
| 38 | Have you ever smoke tobacco products such as cigarettes? *Never =1 Daily=2 Once or twice a week=3 Once a week or more=4 No response=5 Others (specify) _____________* |  |
| 39 | Did you smoke tobacco products such as cigarettes during this current pregnancy? *Yes=1 No=2* |  |
| 40 | Were you admitted anytime in the hospital during current pregnancy? *Yes=1 No=2* |  |
| 41 | If yes to question 64 above, what was the reason for hospital/health centre admission:______________________________________ |  |
| 42 | Did you have malaria attack during the current pregnancy? *Yes=1 No=2* |  |
| 43 | What is the Mid-upper arm circumference (MAUC) of the mother? | _______cm |
| 44 | Family medical history: tick the medical condition that any of your family members has been diagnosed of : Hypertension ( ) Diabetics ( ) Tuberculosis ( ) Congenital malformation ( ) Kidney disease ( ) Heart disease ( ) |  |
| 45 | Personal medical history: tick history of any medical condition you have been diagnosed of: Hypertension ( ) Diabetics ( ) Tuberculosis ( ) Congenital malformation ( ) Kidney disease ( ) Heart disease ( ) Anaemia ( ) HIV ( ) Viral hepatitis ( ) |  |

**SECTION D: Dietary intake of respondent**

Twenty four- hour dietary recall- Please indicate the food consumed in the last 24 hours

**Day of the week: ……………….** **When did you wake up: ……………**

| **Item No.** | **Food/drink Addition** | **Description of food or drink** | **Place taken** | **Time** |
| --- | --- | --- | --- | --- |
|  |  |  |  |  |
|  |  |  |  |  |
|  |  |  |  |  |
|  |  |  |  |  |
|  |  |  |  |  |
| 1. Was the intake usual? (Y/N)   If no. how was it unusual | | | |  |
| 1. Was it a feast day? (Y/N) | | | |  |
| 1. Probe for sickness (Y/N): if yes, did sickness affect your appetite (Y/N)   If yes how? Increase or decrease | | | |  |

| **Session E: Maternal outcomes during labour and delivery** | | |
| --- | --- | --- |
| **S.N** | **Questions** | **Response** |
| **A** | **Maternal outcomes** |  |
| 46 | Delivery type: *Spontaneous vaginal delivery=1 Caesarean Section=2 Forceps delivery=3 Vacuum extraction=4 Other procedures_______________* |  |
| 47 | If delivery was by caesarean section, tick the reason (s) for operative delivery (more than one response is possible): *Breech presentation ( ) Failure to progress in labour ( ) Failure to deliver ( ) Foetal malposition ( ) Placental previa ( ) Other, specify _____________* |  |
| 48 | Presentation of the foetus during delivery: *Cephalic presentation ( ) Breech presentation ( ) Shoulder presentation ( ) Face presentation ( ) Other, specify _____________* |  |
| 49 | Maternal status: *Stable =1 Unstable/deteriorated=2 Died=3* |  |
| 50 | Does the mother have any of the following complications: *Pre-eclampsia ( ) Eclampsia ( ) Antepartum haemorrhage ( ) Postpartum haemorrhage ( ) Shock ( ) Other obstetric complications (specify) __________* |  |
| 51 | Blood pressure measurement before delivery (during the labour process)  (if more than one measurement, write the highest finding) | ------------- |
| 52 | For woman with hypertension, is there proteinuria in urine protein measurement? (if so, write the lab result in the space provided):  *Proteinuria present=1 Proteinuria absent=2* |  |
| 53 | Eclampsia (convulsion in woman with preeclampsia): *Yes=1 No=2* |  |
| 54 | Mother’s body temperature | ________ |
| 55 | Blood pressure immediately after delivery | ________ |
| 56 | Gestational age of the pregnancy during labour/delivery (weeks) | ________ |
| 57 | Time duration from initiation of labour to full cervical dilatation (hours) | ________ |
| 58 | Obstetric induction of labour: *Yes=1 No=2* |  |
| 59 | Premature rupture of membrane: *Yes=1 No=2* |  |
| 60 | Shock *Yes=1 No=2* |  |
| 61 | Episiotomy: *Yes=1 No=2* |  |
